# An acidic patch in the unstructured N‐terminus modulates LSD1 activity

**DOI:** 10.1002/pro.70381

**Published:** 2025-11-13

**Authors:** Franziska Dukatz, Hermann Timofeev, Philipp Schnee, Philipp Rathert

**Affiliations:** ^1^ Department of Molecular Biochemistry, Institute of Biochemistry University of Stuttgart Stuttgart Germany

**Keywords:** biomolecular condensation, catalytic activity, chromatin, chromatin sub‐compartments (CSCs), epigenetic regulation, intrinsically disordered region (IDR), LSD1 (KDM1A), phase separation

## Abstract

Lysine‐specific demethylase 1 (LSD1) plays a crucial role in chromatin organization and gene regulation by removing methyl groups from histone and non‐histone substrates. While its catalytic core is well characterized, the functional contributions of its intrinsically disordered N‐terminal region remain less understood. Here, we identify a conserved acidic patch within this unstructured domain as a key regulator of LSD1 activity. Our findings suggest that this region influences enzymatic efficiency and interactions with regulatory cofactors, shedding new light on the mechanistic control of LSD1 function in epigenetic modulation.

## 1 INTRODUCTION

The traditional models of protein interaction, activation, and structural organization are being reshaped by the growing recognition of liquid–liquid phase separation (LLPS), a fundamental biophysical principle that governs the formation of biomolecular condensates. This process enables the spontaneous emergence of distinct liquid‐like phases within the crowded intracellular environment, giving rise to membrane‐less organelles that play crucial roles in cellular function.

In a nuclear context, LLPS is fundamental to the formation of specialized chromatin sub‐compartments, which serve as hubs for transcriptional regulation, RNA processing, and chromatin organization (Erdel and Rippe 2018). Notable examples include transcriptional condensates that concentrate transcription factors and coactivators (Boija et al. 2018; Papantonis and Cook 2013), P‐granules essential for RNA metabolism (Brangwynne et al. 2009; Smith et al. 2016), and nuclear speckles, which function as reservoirs of splicing factors (Ilık and Aktaş 2022; Zhang et al. 2024). Furthermore, phase separation governs chromatin compartmentalization and higher‐order genome organization (Erdel and Rippe 2018; Gibson et al. 2019; Keenen et al. 2021; Larson et al. 2017; Narlikar 2020; Simon et al. 2007). In many cases, condensate formation is mostly driven by interaction mediated by intrinsically disordered regions (IDRs), which resemble ~30–40% of most eukaryotic proteomes (Lotthammer et al. 2024; Xue et al. 2012).

In recent years, multiple publications reported that the lysine‐specific demethylase 1 (LSD1) is involved in different biomolecular condensation processes, often in concert with coregulatory proteins or nucleic acids (Jia et al. 2021; Senanayaka et al. 2024; Waterbury et al. 2024; Xu et al. 2024). Traditionally, LSD1 is known as a demethylase that removes methyl groups from lysine 4 on histone 3, thereby mediating gene silencing function, often in concert with additional important epigenetic factors, like HDAC (Hakimi et al. 2002), NuRD (Tong et al. 1998), or CoREST (Shi et al. 2005). On the contrary, LSD1 is reported to demethylate H3K9 when interacting with estrogen (Carnesecchi et al. 2017) or androgen receptor (Metzger et al. 2005), which leads to the activation of gene transcription. Further, LSD1 expression is often upregulated in several solid tumors and leukemia and correlated with poor prognosis (Lim et al. 2009; Nagasawa et al. 2015; Pollock et al. 2012). LSD1 was shown to function as a critical cofactor for SNAG domain‐containing transcription factors by forming stable repressive complexes that are essential for their transcriptional silencing activity. It interacts with the SNAG domain of Snail1 and the SNAG domain mimics histone H3 tails and directly recruits LSD1, enabling demethylation of H3K4me1/2 at target promoters, thereby enforcing transcriptional repression (Baron et al. 2011; Chiang and Ayyanathan 2013; Lin et al. 2010). Disrupting the interaction between LSD1 and SNAG domain factors, such as GFI1A/B, impairs their function and differentiation‐blocking capacity in hematopoietic and leukemic cells (Ferrari‐Amorotti et al. 2013; Maiques‐Diaz and Somervaille 2016; Waterbury et al. 2024) highlighting LSD1 as a therapeutic target with several inhibitors in clinical trials already (Noce et al. 2023).

Despite the growing body of research on the role of LSD1 in gene regulation, the functional significance of its capacity for biomolecular condensation and the contribution of its N‐terminal intrinsically disordered region (IDR, residues 1–171) remains incompletely understood. The effect of this IDR on the catalytic activity of LSD1 has not yet been thoroughly investigated. Notably, most structural and biochemical studies have employed truncated versions of LSD1, such as Δ1–122 (Forneris et al. 2007; Pilotto et al. 2015), Δ1–151 (Burg et al. 2016; Stavropoulos et al. 2006), or Δ1–51 (Chen et al. 2013), which still retain portions of the predicted disordered region. A full deletion of the IDR (Δ1–171) has only been studied in the presence of CoREST, which binds to the LSD1 TOWER domain and facilitates nucleosome anchoring (Kim et al. 2020; Pilotto et al. 2015; Yang et al. 2006). However, the activity of the Δ1–171 variant is only observed in the presence of CoREST, and it fails to bind nucleosomes in its absence (Dhall et al. 2020), suggesting a role for the IDR of LSD1 in mediating chromatin engagement and possibly in phase separation. Furthermore, a direct, side‐by‐side comparison of full‐length LSD1 with its truncated forms has not yet been reported (Burg et al. 2016; Chen et al. 2013; Culhane et al. 2006; Forneris et al. 2007; Mimasu et al. 2010; Szewczuk et al. 2007). A recent publication (Senanayaka et al. 2024) highlighted that the N‐terminus of LSD1 enhances the binding of LSD1‐CoREST to nucleosomes and Waterbury et al. (Waterbury et al. 2024) showed that the IDR of LSD1 functions as an autoinhibitory switch that modulates transcription factor binding and AML differentiation. We recently showed that the silencing function of LSD1 is affected by its N‐terminal IDR, presumably due to impaired capacity to form local silencing hubs (Knodel et al. 2024). In support of its functional relevance, we also showed that the IDR can directly incorporate histone peptide substrates and double‐stranded DNA, further highlighting its role as a dynamic interface for chromatin engagement (Knodel et al. 2024).

While intrinsically disordered regions (IDRs) are often associated with phase separation, accumulating evidence suggests that these unstructured domains can also undergo structural transitions upon binding to cofactors, interaction partners, or substrates, thereby contributing directly to binding reactions and functional specificity (Musselman and Kutateladze 2021). Unlike classical lock‐and‐key or induced‐fit models, these interactions often involve context‐dependent folding events, where an initially disordered domain forms higher‐order structures such as α‐helices, β‐sheets, or transient secondary structures in response to molecular recognition (Berlow et al. 2015; Fu et al. 2025; Fuxreiter et al. 2004; Hammes et al. 2009; Oldfield et al. 2008; Wright and Dyson 2009). For example, several transcription factors contain IDRs that transition into ordered structures when engaging with coactivators or DNA, enhancing binding affinity and specificity (Datta et al. 2025; Minezaki et al. 2006). Similarly, scaffold proteins in biomolecular condensates exhibit modular binding domains that remain unstructured until interaction with phase‐separating partners, where they can nucleate multivalent interactions and strengthen condensate integrity (Vazquez et al. 2022). Moreover, the presence of low‐complexity sequences in chromatin‐associated proteins facilitates cooperative binding to nucleosomes, enabling the recruitment of chromatin remodellers or transcriptional machinery in a regulated manner (Gibson et al. 2019).

Despite the growing understanding of the function of LSD1, no systematic study has yet explored how its N‐terminal IDR influences its enzymatic activity. We combined comprehensive in vitro assays with cell‐based approaches to investigate the impact of the N‐terminal IDR on LSD1's demethylation function and gene‐silencing capacity. Through sequential truncation experiments, we demonstrate that the first 160 amino acids (aa) of LSD1 are dispensable for its in vitro demethylation activity. However, we identified a critical acidic patch spanning aa 161–170, which plays a pivotal role in modulating LSD1‐IDR phase separation, enzymatic efficiency, gene silencing function in living cells and the interaction with complex partners, such as HDAC1.

Overall, this study addresses a major gap by systematically dissecting how the IDR of LSD1 influences its biochemical activity and cellular function. Our findings provide new insights into the regulatory mechanisms of LSD1, linking its intrinsically disordered region to enzymatic control and chromatin regulation. This dual role of the unstructured N‐terminal domain of LSD1, both as a driver of phase separation and as a dynamic structural mediator, highlights the functional versatility in cellular regulation. Understanding how these transitions are governed could open new avenues for targeting disordered regions in drug development or manipulating phase‐separating biomolecular complexes for therapeutic applications.

## 2 RESULTS

### 2.1 Regulation of LSD1 enzymatic activity by an acidic IDR subdomain

**FIGURE 1:**
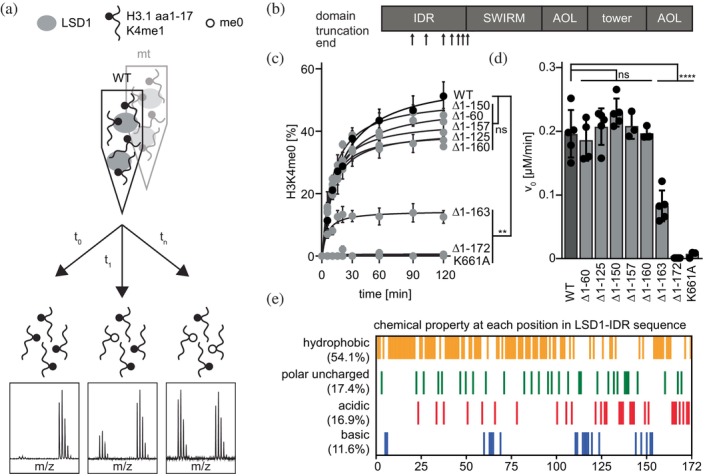
LSD1 harbors an acidic patch at aa 161–170 that drastically impacts catalysis. (a) Schematic representation of LSD1 demethylation analysis. Recombinant LSD1‐His variants expressed in *E. coli* are incubated with H3.1 aa 1–17 K4me1‐Bt peptide (H‐ARTKme1QTARKSTGGKAPR‐Bt‐NH2) and the mass difference of the peptide resulting from the methylation state is detected at different time points by use of MALDI‐ToF. (b) Domain overview of LSD1 and corresponding deletion constructs of LSD1. LSD1 Δ1–60, Δ1–125, Δ1–150, Δ1–157, Δ1–160, Δ1–163, and Δ1–172 denote N‐terminal truncations removing the respective amino acids. (c) Demethylation rates of different LSD1 truncations (1.2 μM) over the course of 120 min together with the exponential fit of each data set. Statistical analysis: Two‐way ANOVA with Dunnett's correction model for multiple comparison (*n* = 3 for K661A, Δ1–157 and Δ1–160; *n* = 5 for all other data, mean ± SEM; ns: not significant, **p < 0.01). (d) Demethylation efficiency shown as initial linear rate of product formation (v_0_) for each demethylation experiment shown in (c). Statistical analysis: Ordinary one‐way ANOVA with Dunnett's correction model for multiple comparison (*n* = 3 for K661A, Δ1–157 and Δ1–160; *n* = 5 for all other data, mean ± SEM; ns: not significant, ****p ≤ 0.0001). (c) Categorization of the chemical property of each amino acid within the IDR of LSD1 (aa 1–172). Hydrophobic: M, L, G, A, P, V, W, I, F. Polar uncharged: Q, S, Y, N, T, C. Acidic: E, D. Basic: R, K, H.

We recently demonstrated that the ability of LSD1 to silence a fluorescent reporter gene in a cellular context (Pinter et al. 2021) is positively influenced by the presence of its N‐terminal intrinsically disordered region (IDR) (Knodel et al. 2024). Sequential N‐terminal truncations resulted in a gradual decline in silencing efficiency (Knodel et al. 2024). While the role of the IDR in LSD1's catalytic activity has only been partially explored, this finding prompted us to further investigate its importance for enzymatic function. To this end, we analyzed the in vitro demethylation activity of recombinant LSD1 variants with progressively shortened N‐termini expressed from bacteria (Figure S1a, Supporting Information), using an H3.1 peptide monomethylated at K4 as substrate (Figure 1a,b). We found that all LSD1 variants with truncations up to amino acid (aa) 160 (Δ1–160) exhibited demethylation activity comparable to wild‐type (WT) LSD1 (Figures 1c,d and S1b), indicating that the distal N‐terminal region does not directly impact catalytic function. As expected, the shortest variant, lacking aa 1–172 (Δ1–172), showed no activity, likely due to disruption of the catalytic SWIRM domain, which begins at aa position 172. Interestingly, the variant lacking aa 1–163 (Δ1–163) displayed a marked reduction in H3K4me1 demethylation, whereas the variant missing only aa 1–160 (Δ1–160) retained WT‐like activity. Notably, these two constructs differ by just three amino acids, all within the unstructured IDR and outside the structured catalytic core. Further analysis of the amino acid composition of the IDR (Figure 1e) revealed a prominent acidic patch between positions 161–170, comprising seven glutamic acid residues at positions 161, 162, 163, 165, 167, 169, and 170. In summary, our systematic analysis of LSD1 variants highlights the functional relevance of an acidic patch within the N‐terminal IDR. ConSurf analysis revealed that the patch exhibits moderate evolutionary conservation and is predicted to be solvent‐exposed, suggesting a potential role in surface‐mediated interactions (Figure S1c).

### 2.2 An acidic patch within the N‐terminal IDR is essential for LSD1 demethylase activity

**FIGURE 2:**
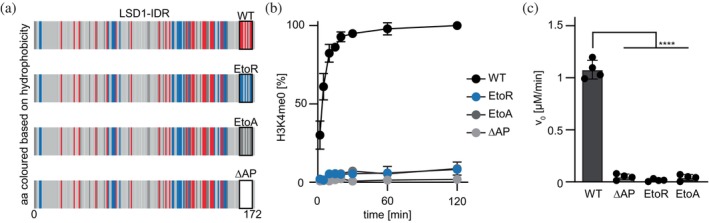
The acidic patch at aa 161–170 is crucial for in vitro demethylation. (a) Presentation of LSD1‐IDR colored based on the hydrophobicity at each position (OHM scale from Sweet and Eisenberg 1983) as gray‐scale with highlights for acidic residues (D, E in red) and basic residues (K, R in blue). Several IDR‐mutants were further investigated and depicted here: Acidic patch EtoR: All E amino acids between aa 161–170 are substituted with R, acidic patch EtoA: All E amino acids between aa 161–170 are substituted with A, ΔAP: In‐frame deletion of aa 161–170. (b) In vitro demethylation efficiency of LSD1 acidic patch mutants (2.2 μM) as depicted in (a) were analyzed by same experimental workflow as depicted in Figure 1a. (c) Demethylation efficiency shown as initial linear rate of product formation (v_0_) for each demethylation experiment shown in a). Statistical analysis: Ordinary one‐way ANOVA with Dunnett's correction model for multiple comparison. Statistical analysis: One‐way ANOVA with Dunnett's correction model for multiple comparison (*n* = 4, mean ± SEM; ****p ≤ 0.0001).

To further dissect the functional relevance of the acidic patch within the IDR, we generated LSD1 variants in which the glutamic acids within aa 161–170 were either substituted with neutral alanine (EtoA) or basic arginine (EtoR) residues. In parallel, we included a LSD1 variant harboring a deletion of the complete acidic patch (ΔAP) (Figure 2a). All respective recombinant variants were successfully expressed and purified from bacteria and CD spectra measurements confirmed that their secondary structure did not markedly deviate from that of the WT enzyme (Figure S2a,b). We then assessed their in vitro demethylase activity, using monomethylated H3K4 peptide as substrate. Strikingly, all variants showed a complete loss of H3K4me1 demethylation (Figures 2b and S2c) in comparison to the WT, despite retaining an intact catalytic center. No protein instabilities could be observed during the demethylation reaction (Figure S2d). This suggests that the acidic patch is not merely accessory but plays an essential role in enzymatic function. One possibility is that the acidic residues transiently interact with the positively charged histone tail, promoting an orientation or proximity that favors efficient catalysis. Alternatively, the patch may stabilize a conformation of the IDR that supports productive substrate binding or allosteric communication with the catalytic core. These results highlight the importance of disordered regions in modulating enzymatic specificity and activity, and suggest that even short linear motifs within IDRs can have profound regulatory effects on chromatin‐modifying enzymes.

### 2.3 The acidic patch covers regions adjacent to catalytic core

**FIGURE 3:**
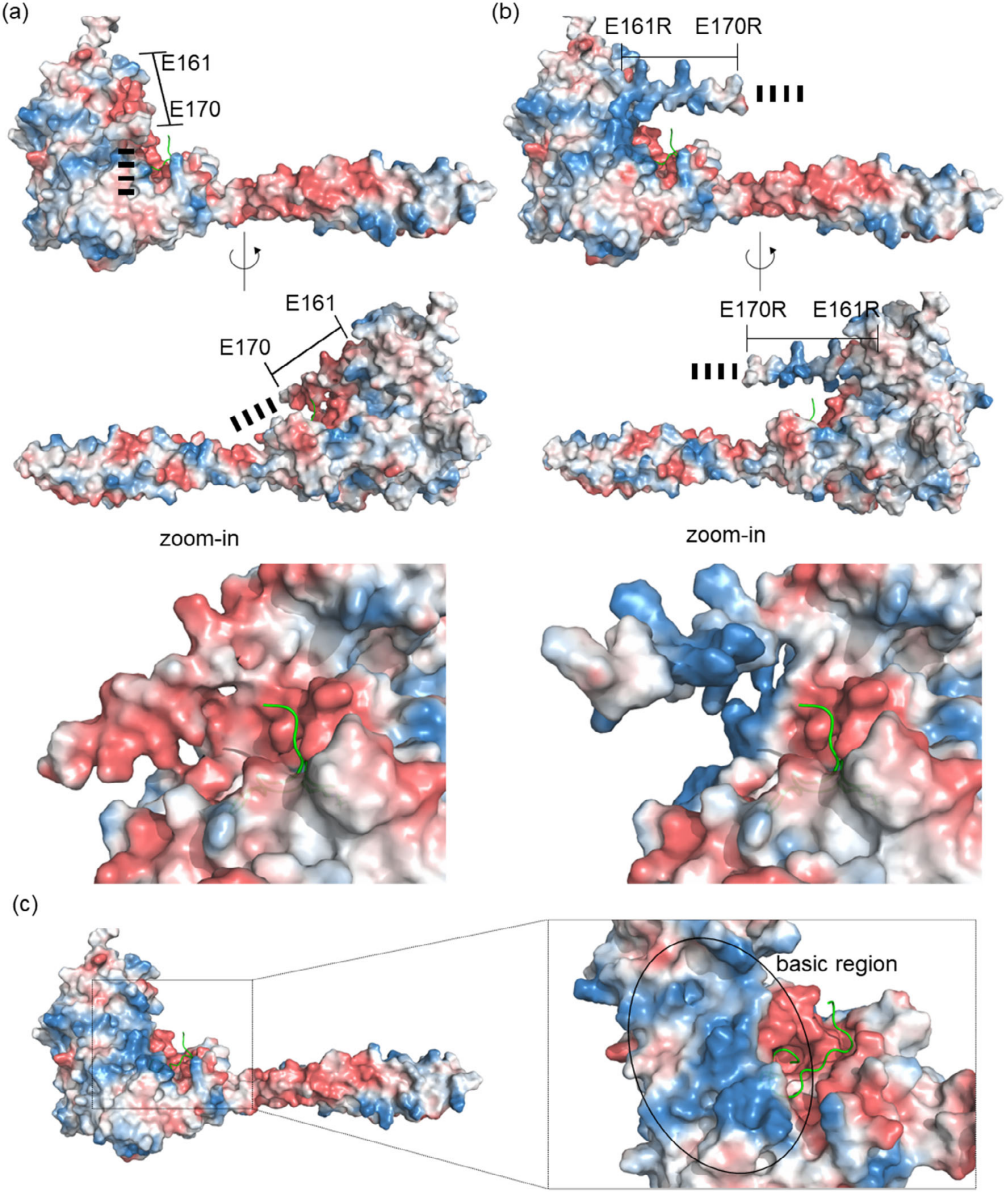
EtoR substitution alters IDR positioning relative to the catalytic center of LSD1. Exemplary AlphaFold prediction of WT‐LSD1 (a) or LSD1 EtoR variant (b) with respective zoom‐ins to visualize catalytic center (bottom). Strong differences could be observed when the glutamic acids within the acidic patch were mutated to arginine, whose distance towards the well‐structured core is drastically increased in comparison to the WT IDR. (c) Overview surface structure of LSD1 aa 170–852 with zoom‐in (right) demonstrates the positively charged region at the surface of LSD1 adjacent to the catalytic site. The charge distribution of all surfaces is displayed as Poisson‐Boltzmann electrostatic potential map (blue = positive, white = neutral, red = negative). The H3.1 peptide aa 1–16 (ARTKQTARKSTGGKAP) is displayed in green.

To gain insight into the functional relevance of the acidic patch in the IDR of LSD1, we employed AlphaFold to model the full‐length structure and examine the spatial relationship between the acidic patch, the histone H3 tail and the LSD1 surface of its structured domains. The AlphaFold prediction for WT LSD1 superimposed exceptionally well with the available crystal structure of the truncated enzyme (Figure S3), providing confidence in the modeled conformations, particularly for the structured core. To ensure the robustness of our observations, each prediction was performed independently three times, generating five models per run (15 models in total per variant), and the results were consistent across all models. Interestingly, in the EtoR variant where glutamic acid residues in the acidic patch were substituted with arginine residues the patch appeared markedly displaced from the structured core relative to the WT (Figure 3a,b). We therefore analyzed the structured core of LSD1 and observed a positively charged region at the surface of LSD1 adjacent to the catalytic site (Figure 3c). In the predicted structure, this region is occluded by the acidic intrinsically disordered region, whose negative charges further enhance the overall acidity at the catalytic pocket in the WT enzyme. In contrast, the charge reversal through arginine substitution appears to disrupt this interaction, likely due to electrostatic repulsion, causing displacement from the corresponding basic region.

These observations support the idea that electrostatic repulsion or attraction within the flexible IDR can induce subtle, but functionally relevant structural rearrangements that ultimately impact enzymatic catalysis.

### 2.4 The acidic patch participates in the phase separation propensity of LSD1 by charge‐dependent condensation

**FIGURE 4:**
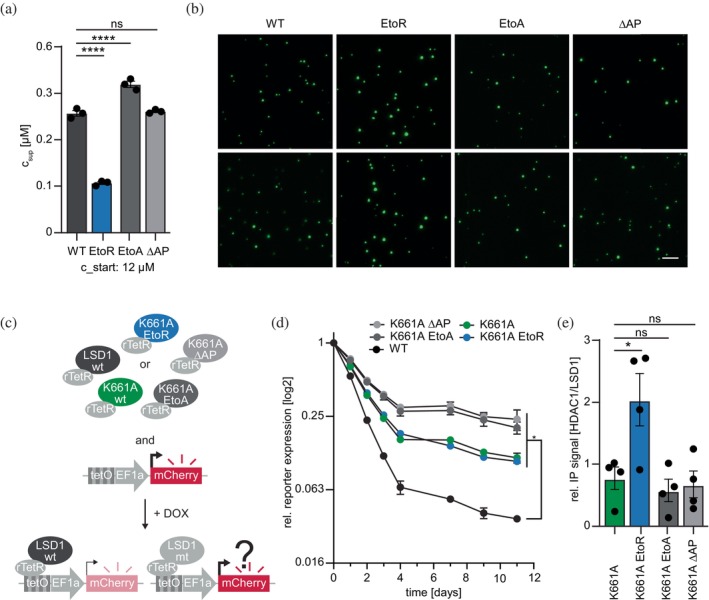
Acidic patch charge inversion increases phase separation of LSD1. (a) In vitro phase separation of IDR‐mVenus‐His recombinantly expressed from bacteria is increased when the Es within the acidic patch are mutated to Rs due to lower protein concentration in the supernatant (c_sup_) after crowding and centrifugation. The strongest increase in condensation could be observed for the EtoR variant, whereas charge neutralization by EtoA slightly reduced phase separation propensity. Statistical analysis: Ordinary one‐way ANOVA with Dunnett's correction model for multiple comparisons (*n* = 3, mean ± SEM; ns: not significant, ****p < 0.0001). (b) Representative pictures of phase‐separated IDR‐mVenus variants. Scale bar = 20 μm. (c) Illustration depicting the rationale of the fluorescent reporter system. Cells of interest are virally transduced with a construct expressing the fluorescent reporter expressed by the EF1a promoter with several tetO binding sites upstream (synthetic promoter). Consecutive transduction with a construct expressing the fusion protein of human LSD1 and the reverse tetracycline repressor protein (rTetR), both with selection cassettes and expression under a constitutive promoter, enables the observation of LSD1 activity in living cells. Upon doxycycline (DOX) treatment, the rTetR‐LSD1 fusion protein is recruited to the synthetic promoter, leading to the suppression of mCherry expression. (d) EtoR substitution within the acidic patch increases the LSD1 K661A reporter silencing. Reporter fluorophore expression was monitored over the course of 11 days in NIH/3T3 reporter cells expressing different LSD1 variants presented in (c). The median reporter expression of cells with DOX‐induced rTetR‐LSD1 recruitment was normalized to reporter cells without rTetR‐LSD1 recruitment. The x‐axis shows the time course. Statistical analysis: Two‐way ANOVA with Dunnett's correction model for multiple comparisons (*n* = 3, mean ± SEM; *p < 0.05). (e) Quantification of HDAC1 over LSD1 signal resulting from co‐immunoprecipitation experiments revealed stronger HDAC1‐LSD1 interaction with the EtoR variant. Statistical analysis: One‐way ANOVA with Dunnett's correction model for multiple comparisons (*n* = 4, mean ± SEM; *p < 0.05). Uncropped Western Blots are shown in Figure S4c. EtoR: All E amino acids between aa 161–170 are substituted with R, EtoA: All E amino acids between aa 161–170 are substituted with A, ΔAP: In‐frame deletion of aa 161–170.

We and others demonstrated that the N‐terminal IDR of LSD1, including the acidic patch, exhibits liquid‐liquid phase separation (LLPS) behavior, but the role of the acidic patch in driving biomolecular condensation remains elusive (Jia et al. 2021; Senanayaka et al. 2024; Waterbury et al. 2024; Xu et al. 2024). Therefore, we generated recombinant LSD1 IDR proteins fused to mVenus (IDR‐mV) with the same EtoA and EtoR substitutions in *E. coli* (Figure S4a). Phase separation propensity was evaluated under macromolecular crowding conditions by pelleting condensates via centrifugation (Bremer et al. 2022) and measuring residual protein concentration in the supernatant (c_sup_). Strikingly, the EtoR variant showed reduced protein levels in the supernatant, indicating enhanced phase separation, whereas the EtoA mutant remained more soluble, suggesting unaffected condensation (Figure 4a). Microscopy further confirmed this trend: the EtoR variant formed larger and more abundant droplets, while the EtoA mutant produced smaller and fewer condensates (Figure 4b). Taken together, these findings demonstrate that the acidic patch within the N‐terminal IDR critically modulates the phase separation behavior of LSD1 in a charge‐dependent manner.

Based on the impact of the acidic patch on biomolecular condensation, we speculate that the respective protein region also impacts the cellular activity of LSD1. LSD1 is a key epigenetic regulator that functions within diverse multiprotein complexes and modulates gene expression in a context‐dependent manner. To gain deeper insights into how the N‐terminal acidic patch contributes to LSD1 function in cells, we utilized the previously established fluorescent reporter system that enables real‐time monitoring of LSD1‐mediated gene silencing in a cellular context (Knodel et al. 2024; Pinter et al. 2021) (Figure 4c). Briefly, it consists of a stably integrated fluorescent reporter gene expressed from a synthetic promoter containing *tetO* binding sites. Upon doxycycline treatment, the rTetR‐LSD1 fusion protein is recruited to the promoter, where it can initiate silencing. This process involves demethylation of H3K4me1/2 and the recruitment of corepressor complexes (Pinter et al. 2021), ultimately leading to reduced reporter fluorescence. In this study, we used a catalytically compromised K661A variant (as in Figure 2a) to isolate non‐enzymatic contributions of LSD1 to gene repression and assess how mutations in the acidic patch affect its silencing capacity. Notably, the K661A mutation does not fully abolish LSD1 activity at the nucleosomal level (Kim et al. 2020). Consistent with earlier studies (Knodel et al. 2024; Pinter et al. 2021), the K661A mutant showed reduced silencing compared to WT LSD1. Notably, both the EtoA mutant and the complete patch deletion (ΔAP) exhibited even more pronounced reduction than the catalytically impaired K661A mutant. Interestingly, the EtoR substitution which replaced the acidic stretch with basic residues, partially rescued the silencing capacity, reaching a similar level as K661A (Figure 4d). To explore the underlying mechanism, we examined the interaction between rTetR‐LSD1 and its co‐repressor HDAC1 using native co‐precipitation assays. Substitution of the acidic patch with arginine residues led to enhanced co‐precipitation of endogenous HDAC1, whereas all other LSD1 variants showed comparable levels of HDAC1 association (Figures 4e and S4c).

Taken together, the data indicate that the acidic patch of LSD1 plays a key role in the enzymatic activity of LSD1 and is essential for its cellular function, most likely by promoting the formation of chromatin sub‐compartments and modulating interactions with coregulatory proteins.

## 3 DISCUSSION

LSD1 (KDM1A) is a multifaceted epigenetic regulator involved in diverse chromatin‐associated processes, including gene repression, enhancer modulation, and transcriptional fine‐tuning. Recent studies have expanded its functional landscape by implicating it in biomolecular condensation events, suggesting that beyond its enzymatic role, LSD1 may also act as a scaffold or organizer of silencing hubs (Waterbury et al. 2024). In line with this view, we and others have proposed that the N‐terminal IDR of LSD1 plays a critical role in mediating its cellular functions, particularly through phase separation and potential co‐condensation with coregulatory partners (Knodel et al. 2024; Waterbury et al. 2024).

In the present study, we aimed to elucidate the dual role of the LSD1 N‐terminal IDR as a mediator of phase separation and as a potential modulator of its enzymatic activity. While the role of IDRs in biomolecular condensation is increasingly appreciated, their direct impact on catalytic efficiency remains less explored. For LSD1 in particular, previous studies offered only fragmentary insight. Waterbury et al. reported that the first 150 amino acids of LSD1 are dispensable for its nucleosome demethylation activity in vitro (Waterbury et al. 2024), but did not extend their analysis to include additional truncations or focus on enzymatic characterization in detail. Similarly, differential nucleosomal binding for LSD1 lacking aa 1–170 was studied in the context of CoREST complexes, leaving open the question about the intrinsic catalytic requirements of LSD1(Kim et al. 2020; Waterbury et al. 2024).

By systematically truncating the N‐terminus of LSD1 and analyzing the enzymatic and phase separation properties, we uncovered a previously unrecognized acidic patch (aa 161–170) within the IDR as a key determinant of LSD1 function. While all N‐terminal truncations up to residue 160 preserved demethylation capacity, deletion or charge‐neutralizing mutations within the acidic patch severely impaired in vitro activity, despite an intact catalytic core. This points to a non‐catalytic yet functionally essential role for the acidic patch, likely in substrate association or stabilization.

Given the basic nature of histone tails, electrostatic interactions between the glutamic acid‐rich region of LSD1 and the lysine‐rich H3 tail seem a plausible mechanism to enhance substrate engagement and catalytic efficiency. Indeed, we demonstrated that the LSD1 IDR can directly incorporate both histone peptides and double‐stranded DNA, indicating its broader role in chromatin association (Knodel et al. 2024). Of note, unstructured regions in other proteins often undergo conformational transitions upon binding their partners (Berlow et al. 2015; Musselman and Kutateladze 2021), suggesting that the LSD1 IDR may similarly adopt specific conformations when interacting with histones or nucleic acids.

Our findings demonstrate that the acidic patch within the unstructured IDR is critical for LSD1 activity in vitro, possibly by facilitating electrostatic interactions with the basic histone tail, thereby ensuring proper substrate positioning. We therefore analyzed the structured core of LSD1 and observed a positively charged region at the surface of LSD1 adjacent to the catalytic site (Figure 3c). In the AlphaFold predicted structure, this region is occluded by the acidic intrinsically disordered region, whose negative charges further enhance the overall acidity at the catalytic pocket in the WT enzyme. In contrast, the charge reversal through arginine substitution appears to disrupt this interaction, likely due to electrostatic repulsion, causing displacement from the corresponding basic region (Figure 3c). Such a conformational rearrangement may disrupt the transient contacts required for effective substrate engagement.

While AlphaFold predictions provide structural hypotheses for potential interaction surfaces, these models are not experimental results and should be interpreted with caution. As such, the analysis presented here is intended as a qualitative framework to support future mechanistic studies rather than a definitive structural conclusion. However, the absence of the acidic patch in the crystal structure and the presence of the basic residues adjacent to the peptide binding grove together support the idea that the acidic patch may serve as a dynamic electrostatic anchor, fine‐tuning the interaction of LSD1 with histone tails and possibly contributing to substrate specificity or chromatin context recognition.

We and others showed recently that the IDR of LSD1 and LSD1 itself forms liquid‐like droplets (Knodel et al. 2024; Senanayaka et al. 2024; Waterbury et al. 2024) and the acidic patch seems to impact this condensation: In sedimentation experiments (Bremer et al. 2022), the EtoR variant exhibited decreased supernatant concentrations resulting from enhanced condensation behavior in comparison to the WT IDR. This enhanced condensation might facilitate the co‐compartmentalization with endogenous corepressors.

Importantly, these effects extend beyond the test tube. Using a previously established fluorescent reporter system (Knodel et al. 2024; Pinter et al. 2021), we examined LSD1 variants harboring both catalytic impairment (K661A) and acidic patch mutations. The EtoA and Δ161–170 variants showed drastically reduced reporter silencing compared to WT or the K661A single mutant, suggesting that the loss of charge or structure in this region compromises LSD1 activity in cells. We interpret this as being consistent with a slight residual enzymatic activity on nucleosomal substrates (Kim et al. 2020) that becomes functionally relevant in the absence of compensatory interactions. The effect of acidic patch deletion therefore reflects a more complete loss of enzymatic function and disrupted co‐compartmentalization of endogenous LSD1 or its cofactors.

In contrast, replacing glutamic acids with arginine (EtoR), thus inverting the local charge, partially rescued silencing, supporting the idea that electrostatic properties in this region influence the capacity of LSD1 to mediate chromatin repression. Additionally, the restored silencing might be supported by enhanced condensate formation as seen in vitro and thereby promoting the co‐recruitment of silencing components. However, such an increased interaction, as shown for HDAC1 in native Co‐IP experiments (Figure 4e), could be directly mediated by local changes in the electrostatic properties. In line with this, the observed effect could be supported by differential substrate interactions. Previous data showed that the IDR is crucial for mediating interactions with LSD1 substrates and nucleic acids (Knodel et al. 2024). While basic substitutions may impair binding to positively charged histone tails, they could simultaneously enhance affinity for negatively charged DNA, potentially compensating for the reduction in catalytic activity through altered condensation behavior, chromatin association and cofactor recruitment (Wang et al. 2018).

We hypothesize, based on our condensation data, that the introduction of positive charges may enhance biomolecular condensation, promoting the formation of larger chromatin sub‐compartments with locally enriched cofactors and substrates and thereby rescuing the additional negative impact of the charge inversion at the acidic patch. These findings align with the emerging understanding that phase separation can modulate enzymatic efficiency by increasing local substrate and cofactor concentrations or promoting conformational dynamics (Boija et al. 2018) and may represent an additional mechanism through which the acidic patch contributes to function.

Taken together, our data strongly support a model in which the acidic patch within the LSD1 IDR serves as a multifunctional module: (1) facilitating charge‐based interactions with either histone substrates to support demethylation or additional coregulatory factors, like HDAC1, and (2) modulating phase separation behavior that may drive the assembly of repressive chromatin sub‐compartments. Given that LSD1 operates in conjunction with other IDR‐containing cofactors, such as HDACs, CoREST, DNMT3A, and PRC2, the potential for cooperative or co‐regulated condensation mechanisms becomes increasingly compelling. Indeed, co‐condensation with these partners could be a key feature of LSD1 biological specificity and efficiency.

In summary, we identify a glutamic acid‐rich acidic patch within the N‐terminal IDR of LSD1 as a critical and previously overlooked determinant of its catalytic and cellular function. These findings highlight the importance of considering disordered regions not as passive linkers but as active, tunable regulators of protein function. Our work opens new avenues for exploring how phase separation and charge‐based interactions orchestrate chromatin dynamics and gene regulation. Our data underscore how subtle changes in charge within disordered regions can rebalance interaction networks and modulate the functional landscape of chromatin‐associated enzymes and provides a foundation for investigating how dysregulation of these properties might contribute to disease.

## 4 MATERIALS AND METHODS

### 4.1 Plasmids

The components for the fluorescent reporter (TSECB6: pMSCV‐tetO‐EF1a‐mCherry‐2A‐Blasti and rTetR‐LSD1: pRRL‐rTetR‐LSD1‐P2A‐Hygro) were generated in (Pinter et al. 2021). The constructs expressing the CCDS of LSD1 (accession number #30627.1) with C‐terminal His tag (pET28‐LSD1‐His) and IDR‐mVenus‐His (aa 1–192 of LSD1) fused to mVenus‐His (pET28‐IDR‐mVenus‐His) were generated in (Knodel et al. 2024).

To generate LSD1 N‐terminal truncations (Δ1–60, Δ1–125, Δ1–150, Δ1–157, Δ1–160, Δ1–163, and Δ1–172), the whole pET‐28‐LSD1‐His construct was PCR‐amplified with Q5 polymerase (NEB, New England Biolabs, USA) and back‐to‐back primers designed to amplify the entire construct in frame while omitting the sequence corresponding to the deleted region.

To generate the point mutation K661A in the pET‐28‐LSD1‐His construct, the whole plasmid was PCR‐amplified in‐frame with Q5 polymerase (NEB, New England Biolabs, USA) and with back‐to‐back primers (LSD1_K661A_F: GCGGTGGTGTTGTGTTTTG, LSD1_K661A_R: GTTAAGGTTGCCAAATCCCATCC) with the forward primer harboring the respective K (AAG) to A (GCG) exchange.

To exchange the acidic patch towards A or E, DNA oligos with respective base exchanges were obtained from IDT (Integrated DNA Technologies, USA) (EtoA: CACCCCCTCAAGCCCCACCTGCTGCAGCTAATGCAAGTGCACCTGCAGCACCATCGGGTGTGGAGGGCGC EtoR: CACCCCCTCAAGCCCCACCTCGTAGACGTAATCGTAGTAGACCTCGTCGGCCATCGGGTGTGGAGGGCGC) and cloned into the in‐frame PCR‐amplified backbone (pET28‐LSD1‐His; LSD1_DelPatch_F: CCATCGGGTGTGGAGG, LSD1_DelPatch_R: AGGTGGGGCTTGAGGG) via NEBuilder HiFi DNA Assembly (NEB) with 25 ng backbone and 50 nM single stranded DNA oligo at 60°C for 10 min followed by 50°C for 1 h. To generate the LSD1 variant lacking the acidic patch at position 161–170 (ΔAP), 50 ng of the in‐frame PCR‐amplified backbone was phosphorylated with the T4 Polynucleotide kinase (NEB, New England Biolabs, USA) and ligated with the T4 DNA ligase (NEB, New England Biolabs, USA) without insert.

After successful integration of respective base exchanges or deletions at the pET28‐LSD1‐His plasmid, they were transferred into the viral pRRL‐LSD1‐constructs. Therefore, corresponding pET28‐LSD1‐His constructs were used as template for Q5‐PCR (NEB) with primers containing suitable overhangs for Gibson assembly. The pRRL‐rTetR‐LSD1‐P2A‐Hygro plasmid was digested with suitable restriction enzymes provided by NEB. Gibson assembly was performed as recommended by NEB.

All cloning steps were validated by Sanger sequencing.

### 4.2 Cell lines

NIH/3T3, Platinum‐E, and Lenti‐X293T cell lines were cultivated in DMEM high glucose media (Sigma‐Aldrich, now Millipore Sigma, USA) supplemented with 10% FBS, 4 mM L‐glutamine, 1 mM sodium pyruvate solution, 10 mM HEPES (pH 7.3), 100 U/mL penicillin, and 100 μg/mL streptomycin in an incubator providing 37°C and 5% CO_2_. All cell lines were cultivated under sterile conditions and tested for mycoplasma contamination on a regular basis.

### 4.3 Cell culture, retroviral transduction, and flow cytometry

NIH/3T3 were used as a model system to analyze LSD1 gene silencing by usage of the fluorescent reporter system, which was first published in (Pinter et al. 2021). Retroviral and lentiviral transduction as well as LSD1 recruitment and silencing analysis were performed as described in (Pinter et al. 2021) and (Knodel et al. 2024). In brief: For retroviral packaging of the reporter construct, 20 μg of plasmid were precipitated for 20 min in HBS buffer (140 mM NaCl, 25 mM HEPES, 0.75 mM Na_2_HPO_4_, pH 7.0) together with 125 mM CaCl_2_ and 10 μg GagPol helper plasmid. The mix was added to a 10 cm dish with Platinum‐E cells growing at 75–85% confluence in supplemented DMEM. After 16 h, the media was exchanged. Viral supernatant was gathered 40–50 h after transfection, filtered through a 0.45 μm filter and added to the target cells at 50–70% confluence. Antibiotic selection was started 2 days after transduction for the reporter construct with 10 μg/mL Blasticidin and kept up for at least 7 days. For packaging of lentiviral rTetR‐LSD1 constructs, desired plasmids were mixed with helper plasmids pCMVR8.74 (pCMVR8.74 was a gift from Didier Trono, Addgene #22036) and pCAG‐Eco (pCAG‐Eco was a gift from Arthur Nienhuis & Patrick Salmon, Addgene #35617) and 3× w/w excess of polyethyleneimine 25 K in serum‐free DMEM. The mix was added to Lenti‐X cells grown in 6‐well disks residing in supplemented DMEM at 75–90% confluence. Media exchanges and transduction of target cells were performed as described for the reporter. Cells expressing pRRL‐rTetR‐LSD1‐P2A‐Hygro were selected with 500 μg/mL Hygromycin. Recruitment of rTetR‐LSD1 was started 12 days after transduction by treatment with 1 μg/mL Doxycycline. mCherry was analyzed using a MACSQuant Vyb flow cytometer (Miltenyi Biotec, Germany) after gating for live and single cells.

### 4.4 Co‐immunoprecipitation and Western blot

10–30 Mio NIH/3T3 cells expressing the respective rTetR‐LSD1 variants were harvested and lysed in 300–900 μL cell lysis buffer (50 mM Tris, 150 mM NaCl, 0.25% Tween 20, pH 7.5) supplemented with cOmplete™ protease inhibitor (Roche). Cells were lysed for 30 min on ice, followed by mild sonication, and further incubated on ice for 30 additional minutes. Lysed cells were centrifuged for 10 min at 16,000*g* and the protein concentration was determined by Bradford assay. 1.5 mg of whole cell protein lysate was incubated with 6 μg TetR Monoclonal Antibody (Clone 9G9, TakaraBio, #631132) for 4–6 h. The antibody‐protein complex was bound by 60 μL protein G Dynabeads™ (Invitrogen) incubated overnight. Beads were washed two times for 5 min with cell lysis buffer. Complexed proteins were eluted for 10 min at 70°C with elution buffer (0.2M Glycine, 0.25% Tween 20, pH 3.0), which was neutralized after elution with 1M Tris/HCl pH 7.5. Prior to antibody incubation, 2% input material was taken.

Proteins were resolved by SDS‐PAGE on a 10% polyacrylamide gel and transferred to an Immobilon‐FL PVDF membrane at 300 mA for 90 min using a wet‐tank blotting system (BioRad) with buffer and ice bucket exchange after 45 min. Proteins were detected after blocking with 5% BSA‐TBS using target‐specific primary antibodies (a‐LSD1, active motif #39186, 1:750; a‐HDAC1, proteintech #10197‐1‐AP, 1:1000 in 1% BSA‐TBST) together with a species‐specific IRDye®680‐coupled secondary antibody. Imaging was performed on an Odyssey® CLx imaging system (LI‐COR).

### 4.5 Protein expression and purification

C‐terminal His‐tagged LSD1 variants and IDR‐mVenus variants were expressed and purified as described in (Knodel et al. 2024). In short, *E. coli BL21‐CodonPlus (DE3)‐RIL* cells were transformed with the desired plasmid and plated on LB agar with 35 μg/mL Chloramphenicol and 50 μg/mL Kanamycin and grown overnight. Subsequently, selection media was inoculated with one colony and the starter culture was cultivated at 37°C, 150 rpm for 6 h. 500 mL LB media with respective antibiotics was inoculated with 6 mL of starter culture and cultivated at 37°C, 150 rpm until OD_600_ = 0.6. Protein expression was induced by the addition of 200 μM IPTG and overexpression was performed at 17°C, 150 rpm for 14 h. Cells were harvested at 5000*g* for 15 min, 4°C. Pellets were washed once in 30 mL STE buffer (100 mM NaCl, 10 mM Tris HCl pH 8, 1 mM EDTA) and frozen at −20°C until use. For purification, pellets were resuspended in 30 mL sonication buffer (30 mM KPI‐buffer pH 7.2, 0.2 mM DTT, 500 mM KCl, 1 mM EDTA, 10% glycerol, 20 mM imidazole) with a protease inhibitor and lysed by sonication using an EpiShear Probe Sonicator (Active Motif, USA). The lysate was cleared by centrifugation and filtration through a 0.45 μm CHROMAFIL GF/PET‐45/25 filter (MACHEREY‐Nagel, Germany). Affinity chromatography was performed using an NGC™ Chromatography system (BIO‐Rad, USA) and purification columns packed with Ni‐NTA superflow beads (Clontech, now TAKARA Bio, USA). Proteins were eluted in elution buffer (30 mM KPI‐buffer pH 7.2, 500 mM KCl, 0.2 mM DTT, 1 mM EDTA, 10% glycerol, 220 mM imidazole) and subjected to dialysis into storage buffer (20 mM HEPES pH 7.2, 200 mM KCl, 0.2 mM DTT, 1 mM EDTA, 10% glycerol). Aliquots were snap‐frozen and stored at −80°C. After successful purification, total protein purity and amount were validated by standard SDS‐PAGE followed by Coomassie brilliant blue staining.

### 4.6 Circular dichroism spectroscopy

The protein folding was analyzed by circular dichroism spectroscopy using a J‐815 CD spectrophotometer (JASCO Corporation, Tokyo, Japan). 10 μL of each protein present in dialysis I storage buffer (20 mM HEPES pH 7.2, 200 mM KCl, 0.2 mM DTT, 1 mM EDTA, 10% glycerol) was diluted in 30 μL of 200 mM KCl. The spectra were collected at 20°C using a 0.1 mm cuvette in a wavelength range between 190 and 250 nm using a scanning speed of 50 nm/min, a bandwidth of 1 nm, and a data integration time of 2 s. For each sample, 40 scans were collected and averaged.

### 4.7 LSD1 demethylation assay

Equal amounts of recombinant LSD1 variants (1.2 or 2.2 μM) were incubated at 37°C with 12 μM H3.1 1–17 K4me1‐Bt peptide (H‐ARTKme1QTARKSTGGKAPR‐Bt‐NH2, Intavis, Germany) in HDM buffer (21.43 mM Tris–HCl pH 8.5, 21.43 mM KCl, 2.14 mM MgCl_2_, 0.8 mg/mL BSA) and 11.54% of the total reaction was protein/dialysis buffer. Samples were taken at different time points and processed for MALDI‐ToF (Bruker, Germany) as described in Knodel et al. (2024).

### 4.8 In vitro droplet formation and c_sup_ determination

6 μM of respective IDR‐mVenus variants in comparison to mVenus alone was incubated for 30 min under crowding conditions (20% PEG‐8000, 40 mM TRIS–HCl pH 7.5) at 20°C. After crowding, samples were centrifuged for 5 min at 21,500*g*. Supernatant was transferred and mV fluorescence was determined by using EnSpire Multimode plate reader (PerkinElmer, USA).

### 4.9 AlphaFold 3 structure prediction

The 3D structure of LSD1 WT (PDB: 2V1D) (Forneris et al. 2007) or the acidic patch mutated LSD1 variant (E to R) in complex with the H3.1 tail sequence (ARTK(me2)QTARKSTGGKAPRKQLATKAARKSAP) and CoREST (PDB: 2V1D (Forneris et al. 2007) was predicted by AlphaFold 3 to identify differences in the hypothetical positioning of the acidic patch. AlphaFold 3 prediction was performed 3 times in total. All structures were visualized using PyMOL (3.1.1). The surface charge distribution was represented as a Poisson–Boltzmann electrostatic potential map to illustrate charge variations across the molecular surface.

## AUTHOR CONTRIBUTIONS

**Franziska Dukatz:** Writing – review and editing; visualization; data curation; formal analysis; writing – original draft. **Hermann Timofeev:** Methodology. **Philipp Schnee:** Writing – review and editing; visualization. **Philipp Rathert:** Conceptualization; writing – original draft; writing – review and editing; funding acquisition; project administration; supervision; resources.

## CONFLICT OF INTEREST STATEMENT

The authors declare no conflicts of interest.

## Supporting information


**Figure S1.** (connected to Figure 1): (a) Comparable protein amounts.
**Figure S2.** (connected to Figure 2): (a) Representative SDS‐PAGE.
**Figure S3.** (connected to Figure 3): (a) Superposition of LSD1 crystal.
**Figure S4.** (connected to Figure 4): (a) Representative SDS‐PAGE.

## Data Availability

The data that support the findings of this study are available from the corresponding author upon reasonable request.
