# Supplementary material for: An acidic patch in the unstructured N‐terminus modulates LSD1 activity
Source: Protein Sci. 2025 Nov 13;34(12):e70381. doi: 10.1002/pro.70381 (PMC12612597; doi:10.1002/pro.70381)
Supplement: Supplementary file 1 — Figure S1. (connected to Figure 1): (a) Comparable protein amounts. Figure S2. (connected to Figure 2): (a) Representative SDS‐PAGE. Figure S3. (connected to Figure 3): (a) Superposition of LSD1 crystal. Figure S4. (connected to Figure 4): (a) Representative SDS‐PAGE. [file PRO-34-e70381-s001.pdf]

Supplementary Figures

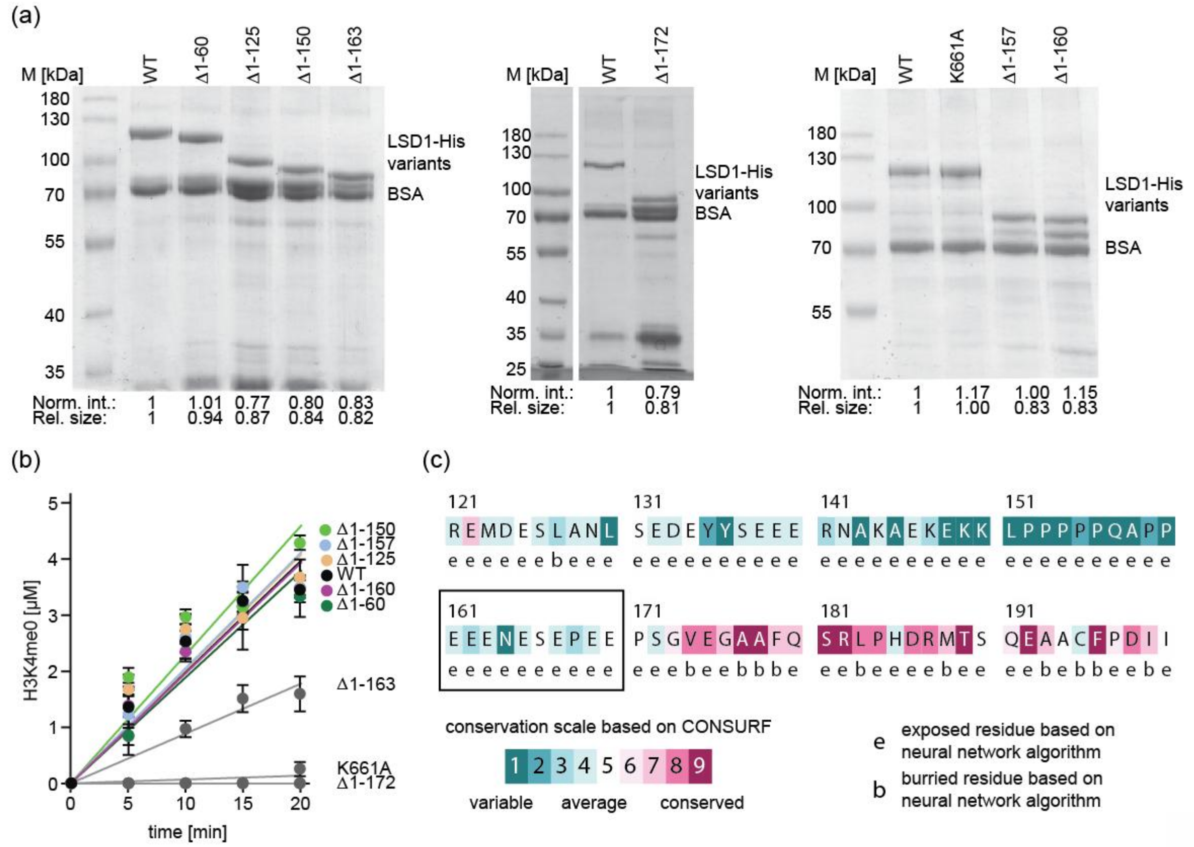

Supplementary Figure 1 (connected to Figure 1): (a) Comparable protein amounts of different recombinant LSD1 variants for demethylation analysis: After demethylation reaction, equal reaction amounts were loaded on 12% polyacrylamide gel to verify same protein amounts (Norm. int.) within each reaction in respect of each protein size (Rel. size). (b) Averaged linear regression over the first 20 min of demethylation reaction shown in Fig. 1c, d. presented as H3K4me0 in µM over time [min] (n = 3 for K661A, Δ1-157 and Δ1-160; n=5 for all other data, mean ± SEM). LSD1 Δ1-60, Δ1-125, Δ1-150, Δ1-157, Δ1-160, Δ1-163 and Δ1-172 denote N-terminal truncations removing the respective amino acids. (c) Protein sequence alignment of LSD1 aa 121-200 generated with CONSURF highlighting variable or conserved amino acid residues within this region. The acidic patch is marked by a box. The letters e and b below indicate the accessibility of each residue as predicted by the network.

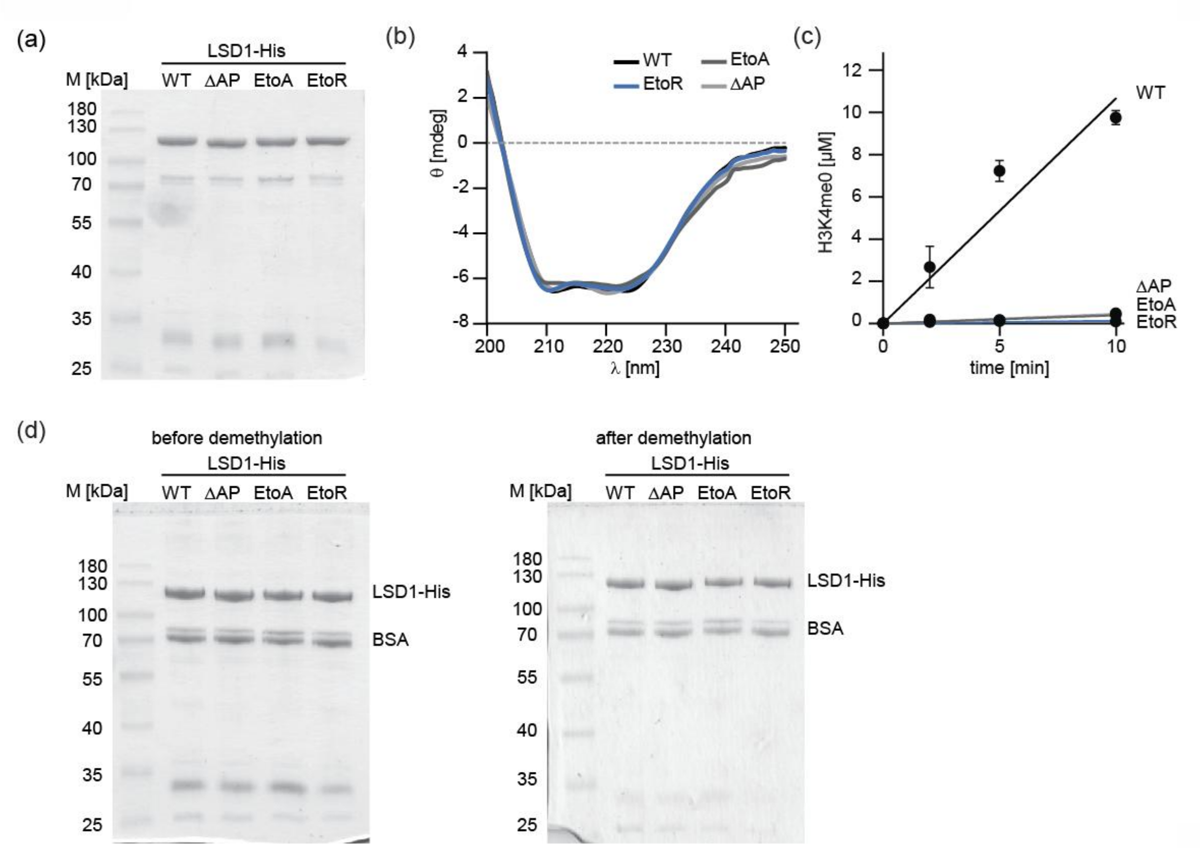

Supplementary Figure 2 (connected to Figure 2): (a) Representative SDS-PAGE picture of recombinant LSD1-His WT and respective acidic patch mutant after purification. (b) CD-spectra of recombinant LSD1-His WT and respective acidic patch mutant demonstrating comparable protein folding. (c) Averaged linear regression over the first 10 min of demethylation reaction shown in Fig. 2b, c presented as H3K4me0 in µM over time [min] (n=4, mean ± SEM). (d) LSD1 demethylation reaction before and after incubation for 60 min on 37°C. EtoR: E amino acids between aa 161-170 are substituted with R, EtoA: E amino acids between aa 161-170 are substituted with A, ΔAP: in-frame deletion of aa 161-170.

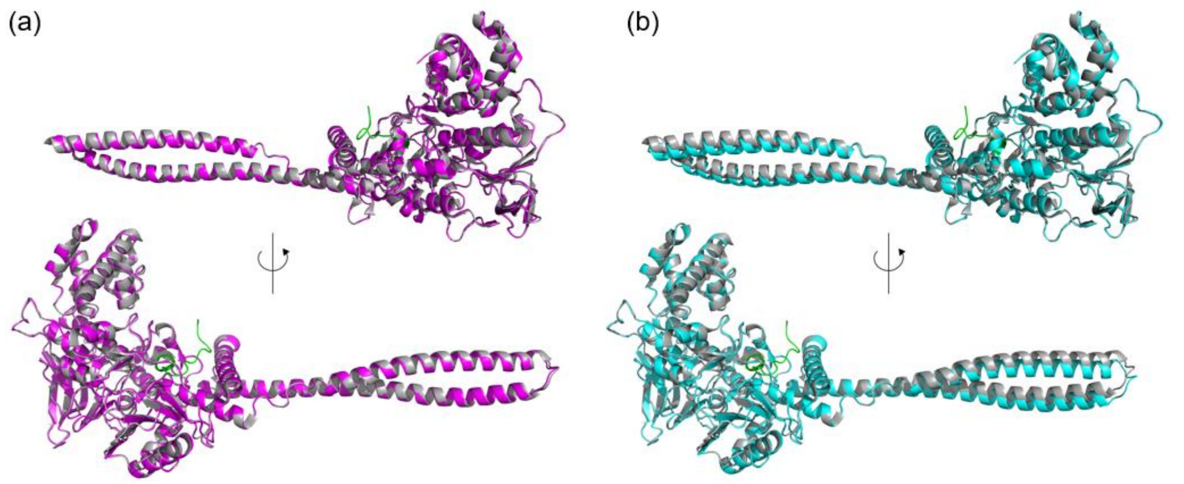

Supplementary Figure 3 (connected to Figure 3): (a) Superposition of LSD1 crystal structure in grey (PDB: 2V1D) and 3D structure of LSD1 WT in magenta predicted by AlphaFold together with H3.1 aa 1-16 (ARTKQTARKSTGGKAP) in green. (b) Superposition of LSD1 crystal structure in grey (PDB: 2V1D) and 3D structure of LSD1 EtoR mutant in turquoise predicted by AlphaFold together with H3.1 aa 1-16 (ARTKQTARKSTGGKAP) in green.

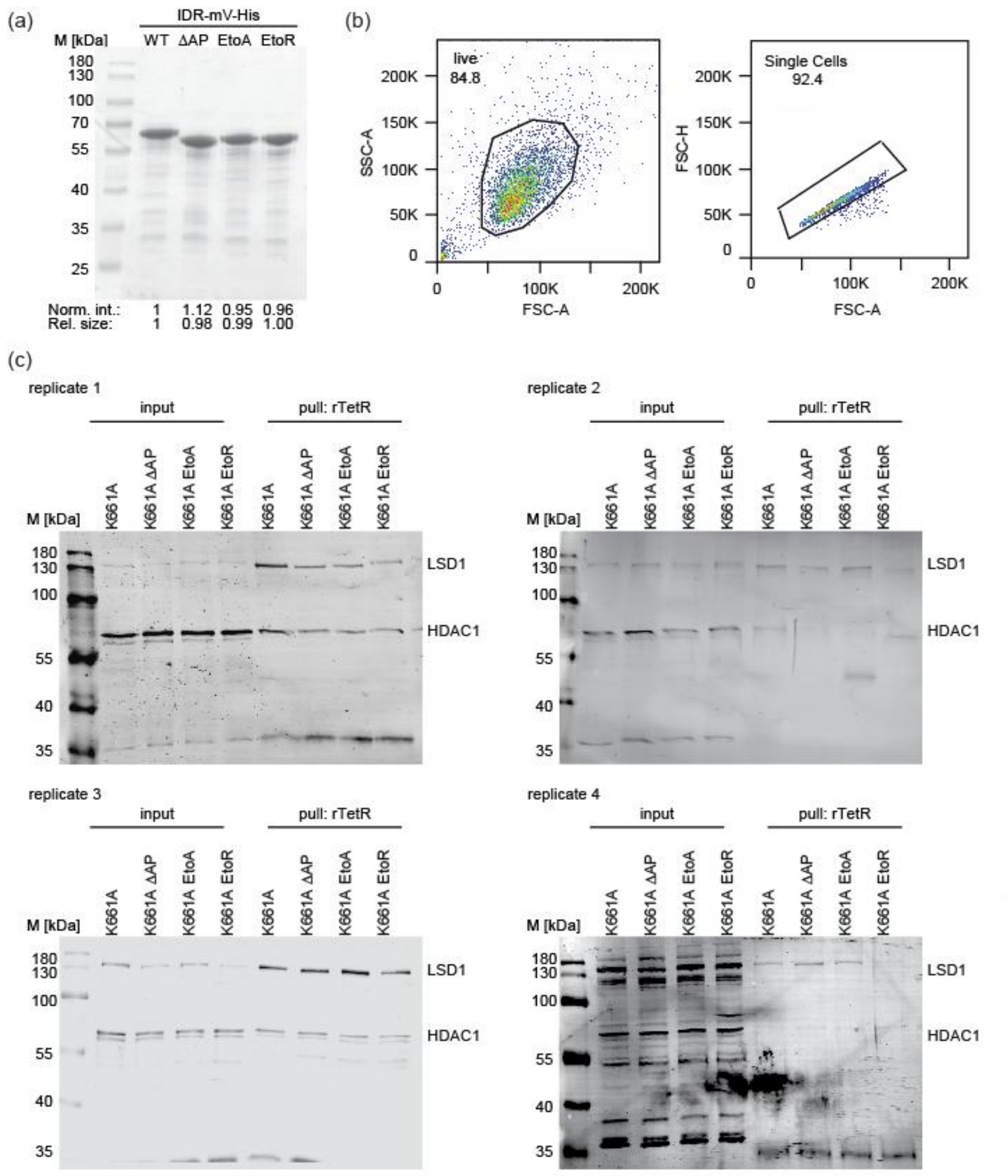

Supplementary Figure 4 (connected to Figure 4): (a) Representative SDS-PAGE picture of recombinant IDR-mV WT and respective acidic patch mutants validating comparable protein amounts (Norm. int.) in respect of each protein size (Rel. size). (b) Flow cytometry gating strategy. All events were analysed in scatterplots of sideward-scatter-area (SSC-A) over forward-scatter-area (FSC-A) to detect all living cells (live, % of total). Duplet cells were excluded in scatterplots of forward-scatter-area over forward-scatter-height (Single Cell, % of total). (c) Complete Western Blot images from four independent Co-immunoprecipitation experiments. Respective rTetR-LSD1 variants were immunoprecipitated using an anti-TetR antibody. Co-

precipitated HDAC1 was detected by immunoblotting. Input lysates (0.4%) were included as controls. The strong additional bands in the input samples in replicate 4 resulted from repetitive reprobing with additional primary antibodies. EtoR: E amino acids between aa 161-170 are substituted with R, EtoA: E amino acids between aa 161-170 are substituted with A, ∆AP: in-frame deletion of aa 161-170.
